# Draft Genome Sequence of *Moraxella catarrhalis* Type Strain CCUG 353^T^

**DOI:** 10.1128/genomeA.00552-16

**Published:** 2016-06-16

**Authors:** Hedvig E. Jakobsson, Francisco Salvà-Serra, Kaisa Thorell, Lucia Gonzales-Siles, Fredrik Boulund, Roger Karlsson, Per Sikora, Lars Engstrand, Erik Kristiansson, Edward R. B. Moore

**Affiliations:** a Department of Infectious Diseases, Sahlgrenska Academy, University of Gothenburg, Gothenburg, Sweden; b Culture Collection University of Gothenburg (CCUG), Sahlgrenska Academy, University of Gothenburg, Gothenburg, Sweden; c Microbiology, Department of Biology, University of the Balearic Islands, Palma de Mallorca, Spain; d Department of Microbiology, Tumor and Cell Biology, Karolinska Institute, Stockholm, Sweden; e Department of Mathematical Sciences, Chalmers University of Technology, Gothenburg, Sweden; f Nanoxis Consulting AB, Gothenburg, Sweden; g Integrative Clinical Genomics, Clinical Diagnostics, SciLifeLab, Stockholm, Sweden; h Laboratory Medicine, Sahlgrenska University Hospital, Gothenburg, Sweden

## Abstract

*Moraxella catarrhalis* is a Gram-negative commensal and pathogenic bacterium found in the human respiratory tract. It is associated with otitis media and respiratory tract infections. Here, we report the draft genome sequence of *M. catarrhalis* type strain CCUG 353^T^, composed of 18 contigs and a total size of 1.89 Mb.

## GENOME ANNOUNCEMENT

*Moraxella catarrhalis* is a Gram-negative, aerobic, nonencapsulated diplococcus commonly found in the human upper respiratory tract, especially in children (1). It is considered to be commensal and pathogenic (2) and it has been related with otitis media and respiratory tract infections (e.g., sinusitis and pneumonia). It is composed of two distinct lineages with different virulence potential (3). One lineage is considered serosensitive with moderate virulence, while the other lineage is considered seroresistant with several virulence traits (3). *M. catarrhalis* was first described in 1896 as “*Mikrokkokus catarrhalis*” (4) and later reclassified (5) in the genus *Moraxella* (6). It is microscopically similar to *Neisseria meningitidis* and *Neisseria gonorrhoeae* but phylogenetically related to the genus *Acinetobacter* (7) within the class *Gammaproteobacteria* (8). 

*M. catarrhalis* CCUG 353^T^ (= Catlin Ne11 = ATCC 25238^T^ = NCTC 11020^T^) was cultivated at 37°C with 5% CO_2_ on Columbia agar base plus 5% horse blood. A fresh, pure-culture biomass was lysed in cell lysis buffer (1 mM Tris, 0.1 mM EDTA, pH 8.0), supplemented with Triton 1.2% (wt/vol), lysozyme (20 mg/ml), and proteinase K (1 mg/ml), at 56°C for 1 h. Genomic DNA was isolated, using a MagNA Pure Compact Nucleic Acid Isolation Kit version I (Roche Diagnostics, Mannheim, Germany). DNA was sequenced with an Illumina MiSeq instrument (SciLifeLab, Stockholm, Sweden), generating 1,439,812 paired-end reads of 300 bp, yielding a total of 432 Mb. The DNA was also sequenced with an Ion Torrent PGM (Sahlgrenska University Hospital, Gothenburg, Sweden), generating 872,848 single reads with a mean read length of 279 bp and yielding a total of 244 Mb. The two sets of reads were trimmed and used together to make a hybrid *de novo* assembly with CLC Genomics Workbench version 8 (CLC bio, Aarhus, Denmark). Assembly quality was assessed using QUAST version 3.1 (9). Trimming of the sequence reads left 1,437,467 Illumina paired-end reads with an average of 211 bp (70% of initial sequence data) and 872,845 Ion Torrent single reads with an average of 214 bp (77% of initial sequence data). In total, 2,310,312 reads were used for the assembly, producing a final assembly length of 1,886,586 bp, consisting of 18 contigs. The longest contig measures 457,892 bp, the *N*_50_ is 290,242 bp, and the average genome coverage of the assembly is 260×. The G+C content of the genome sequence is estimated at 41.5%. The genome sequence was annotated using the NCBI Prokaryotic Genome Annotation Pipeline (PGAP) (10), and 1,685 coding sequences and 44 tRNAs were identified. Analysis of the genome sequence for the presence of CRISPR-Cas systems (11), with CRISPRFinder (12), confirmed the presence of a CRISPR region formed by two arrays of spacer repeats that contained 25 and 11 spacers, respectively.

### Nucleotide sequence accession numbers.

This whole-genome shotgun project has been deposited at DDBJ/ENA/GenBank under the accession number LWAH00000000. The version described in this paper is the first version, LWAH01000000.
